# Trade-off between fertility and predation risk drives a geometric sequence in the pattern of group sizes in baboons

**DOI:** 10.1098/rsbl.2017.0700

**Published:** 2018-03-07

**Authors:** R. I. M. Dunbar, Padraig MacCarron, Cole Robertson

**Affiliations:** 1 Department of Experimental Psychology, University of Oxford, South Parks, Oxford OX1 3UD, UK; 2 Department of Computer Science, Aalto University, Espoo FI-00076, Finland

**Keywords:** social organization, fission, fertility, predation risk, evolutionarily stable strategy

## Abstract

Group-living offers both benefits (protection against predators, access to resources) and costs (increased ecological competition, the impact of group size on fertility). Here, we use cluster analysis to detect natural patternings in a comprehensive sample of baboon groups, and identify a geometric sequence with peaks at approximately 20, 40, 80 and 160. We suggest (i) that these form a set of demographic oscillators that set habitat-specific limits to group size and (ii) that the oscillator arises from a trade-off between female fertility and predation risk.

## 1. Introduction

Mammal social group size represents a trade-off between the costs and benefits of sociality [1] subject to a limit set by habitat productivity [2]. For most birds and mammals, one of the major benefits of living in groups is protection from predators [3–7], with the benefits typically increasing with group size. The costs arise from a combination of competition for access to food [2,8] and the social stresses created by living in close proximity [9–13]. These costs are invariably reflected in female fertility, such that fertility correlates negatively with group size across mammals [9,14] (see the electronic supplementary material). The difficulty for those mammalian taxa that live in bonded social groups [15] (as opposed to more casual aggregations) is that there are structural constraints on a group's ability to shed members when the group becomes too large; instead, the group has to continue growing until it is large enough to fission. Rather than maintaining a steady state through ‘trickle emigration’ (individual animals emigrating on their own), groups will oscillate in size across a range set by the minimum acceptable group size [2].

We here explore how fertility and predation risk intersect to determine group size across habitats in an intensely social primate, baboons (genus *Papio*). We first use cluster analysis to ask whether the distribution of group sizes is unimodal or multi-modal. A unimodal distribution would suggest that groups are randomly distributed around a taxon-typical mean, whereas a multi-modal distribution, especially if those modes are fractally related, would suggest a regular pattern of fission. We then ask whether female fertility varies systematically with group size and, if so, whether this might explain the distribution of group sizes.

## 2. Material and methods

We limited our analysis to the four ‘woodland’ species (*P*. *anubis*, *P. cynocephalus*, *P. ursinus* and *P. papio*) because *Papio hamadryas* has a radically different harem-based social system. We comprehensively searched the literature for census data on group sizes. The criteria for inclusion are summarized in the electronic supplementary material*.* This yielded a total of 410 groups across 45 study sites in 13 countries (the data are given in the electronic supplementary material, Dataset1).

We used maximum-likelihood methods [16] to fit a set of common distributions, and identified the best model using AIC. We identified cluster mean values from this, and then checked these using a different approach (Jenks natural breaks algorithm). For details, see the electronic supplementary material.

Female fertility rates for 12 individual baboon groups are taken from [10], with additional data for one group each for *P. anubis* and *P. ursinus*, and two population means for *P. papio* (for details and data, see the electronic supplementary material, and Dataset2). Environmental data for these habitats are from [10,17].

## 3. Results

**Figure 1.**
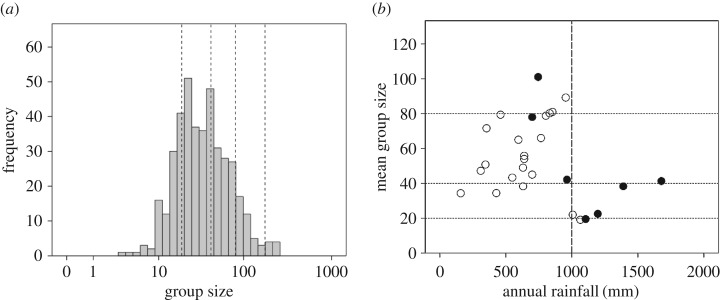
(*a*) Distribution of social group size in baboons. Dashed vertical lines indicate the cluster means, averaged for the two clustering algorithms (see text for details). (*b*) Mean group size for individual baboon populations, plotted against annual rainfall. Open symbols: estimated predator density less than 0.25 km^−2^; solid symbols: predator densities greater than 0.25 km^−2^ (see text for details). Horizontal dotted lines indicate boundaries of the 20/40 and 40/80 oscillators. Vertical dashed line demarcates the apparent phase shift at 1000 mm rainfall. Source: [18].

The distribution of *Papio* group sizes is highly skewed, with a mean of 43.6 ± 36.65 s.d. and a range of 3–247 (figure 1*a*). Applying maximum-likelihood estimation to the raw data, AIC finds that the distribution is most likely made up of four Poisson distributions (table 1). Both the maximum likelihood estimation and Jenks algorithms give similar cluster means (electronic supplementary material, table S1), with averaged values at 19.1, 42.1, 80.5 and 175.7 (figure 1*a*). Electronic supplementary material, figure S1 plots the four theoretical clusters on the actual distribution of individual group sizes. Both series have a mean scaling ratio of 2.1, suggesting a pattern indicative of binary fission. Jenks also identifies three or four as the optimal number of clusters for all four species individually, with cluster means that are close to those found for the combined sample (electronic supplementary material, table S2).

**Table 1.** AIC values for the models describing the distribution of baboon group sizes. The best-fit model is shown in bold.

distribution	AIC
power law	4632.7
exponential	4006.5
truncated power law	4099.1
Weibull	3978.1
Gaussian	4061.6
lognormal	3914.6
geometric	4045.3
negative binomial	3967.5
Poisson (single)	11829.6
*Compound Poisson:*	
*n* = 2	5506.5
*n* = 3	3874.7
***n* = 4**	**3086**.**1**
*n* = 5	3088.1
*n* = 6	3090.2
*n* = 7	3092.3

We interpret the geometric sequence formed by these means (approx. 20, 40, 80, 160) as a set of three demographic oscillators (20/40, 40/80, 80/160). In each case, a group will oscillate in size over time between a pair of limits (e.g. 20–40): natural growth rates cause a group to increase in size through births until it fissions at around the upper value to return back to the lower value, and begins once more to grow. The data suggest that populations characteristically occupy one, and only one, oscillator at any given time (electronic supplementary material, figure S2).

Why might there be several distinct oscillators? We suggest that the preferred oscillator is set by local predation risk, combined with the impact of group size on fertility. As the largest cluster (approx. 160) is extremely rare (only 4% of groups are larger than 120), we focus here on the lower two pairings (one with attractors at approx. 20 and approx. 40, the other with attractors at approx. 40 and approx. 80).

Predation risk is a composite of predator density (the likelihood of encountering a predator) and the density of refuges (large trees) in which to escape from predators [18,19]. We use annual rainfall as a well-established proxy for tree cover [20]; we calculate predator density from separate climate envelope models for leopard and lion (the two principal predators of baboons [3]), as given by Bettridge *et al*. [17]. Figure 1*b* indicates that baboon group size varies between 40 and approximately 80 in drier habitats (low tree cover) and then plummets to between 20 and 40 in habitats with more than 1000 mm rainfall (high tree cover). There is some suggestion that, within each set, populations occupying habitats with high predator densities (more than 0.25 leopards and lions per km^2^) live in larger groups than those in low predator density habitats. Comparison of goodness of fit across different rainfall cutoffs indicates that there is a clear transition at approximately 1000 mm rainfall (electronic supplementary material, figure S3).

**Figure 2.**
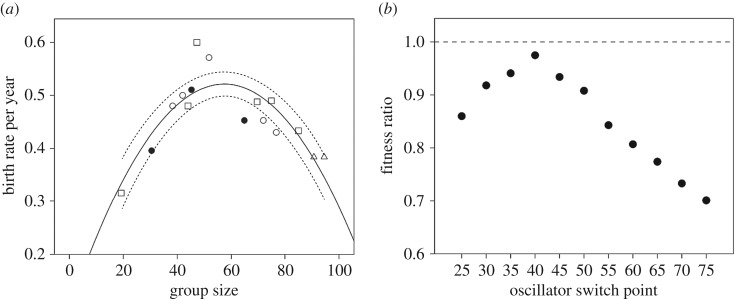
(*a*) Mean fertility (births per adult female per year) for individual baboon groups, plotted against group size. Filled circles: *P. anubis*; open circles: *P. cynocephalus;* squares*: P. ursinus*; triangles: *P. papio*. The best-fit least-squares regression has a quadratic form (solid line, with 95% CI of mean indicated by dotted lines). (*b*) Ratio of payoffs (smaller/larger) for different possible oscillator pairs. Payoff is the number of offspring produced in an average 13-year reproductive lifespan, given the fertility schedule in (*a*) as group size changes over time. The switch point is the dividing group size between the two oscillators: a switch point at 30 indicates an oscillator pair of 20–30 versus 30–80. For details, see the electronic supplementary material.

To explore the impact of fertility, we plot mean birth rate against group size for 16 individual baboon groups (figure 2*a*). The data are best explained by a quadratic regression (*F*_2,13_ = 15.73, *r*^2^ = 0.708, *p* = 0.0003; linear: *F*_1,14_ = 0.18, *r*^2^ = 0.012, *p* = 0.682; for details, see the electronic supplementary material). We checked whether this might be due to environmental conditions by regressing birth rate against temperature (an index of habitat quality: see the electronic supplementary material) and plotting the residuals against group size; the results are essentially the same (electronic supplementary material, figure S4). AIC model comparison indicates that group size is a more important determinant of fertility than environmental quality (electronic supplementary material, table S3).

To determine whether a 20–40/40–80 split is evolutionarily stable, we calculated the payoff to a female in a given oscillator as the number of offspring born over an average reproductive lifespan (13 years) with the fertility schedule in figure 2*a* and progressive fertility-determined growth in group size over time (for details, see the electronic supplementary material). Figure 2*b* plots the payoff ratio (smaller/larger payoff) across the range of possible oscillator pairs. It is evident that only a 20–40/40–80 pairing comes close to equality of payoffs. Deviations away from this progressively favour one over the other rather than a balanced strategy set.

## 4. Discussion

The regular patterning in the distribution of baboon group sizes suggests that there is an underlying geometric pattern that makes certain values (approx. 20, approx. 40, approx. 80 and approx. 160) particularly common. This signal can be detected not just in the sample as a whole but also in all four species-specific sub-samples. The fact that groups form a scaled series is unexpected, because it suggests that certain group sizes are more stable demographically than others. There is no obvious ecological reason why this should be so; nor can it be attributed to phylogeny (i.e. differences between species) because all the species have essentially the same group size signature.

We suggest that these values represent a set of demographic oscillators within which group size cycles, with the lower value for each oscillator determined by the local predation risk (figure 1*b*) and the upper value by the minimum size for fission to yield the lower value at the end of the cycle (a group must be at least twice the size of the minimum daughter group before fission can occur [2]) combined with the impact of group size on fertility (figure 2*a*). The limited evidence on size at fission supports this: baboons in a high predator density habitat in East Africa fissioned at a mean size of approximately 65 (population mean group size 50.7; *N* = 51), whereas in a low predator density habitat in South Africa they did so at a mean size of approximately 32 (mean group size 22.4; *N* = 61) [21].

Figure 2*b* suggests that the 20–40/40–80 pairing is a stable strategy set. Its payoff ratio is the only one close to parity; all alternative transition points yield ratios that increasingly favour one oscillator over the other, and would result in the group size distribution being dominated by the more advantageous oscillator. The fact that the payoff ratio optimizes at a transition point (approx. 40) very close to mean genus group size (43.6) strongly suggests that this is evolutionarily stable: payoffs and frequencies are in balance. This particular split means that females face much the same fertility regime across their reproductive lives irrespective of which oscillator they adopt, the difference being only whether they have low fertility early or late in their reproductive careers.

Which oscillator occurs in a particular location seems to depend entirely on the local predation risk. Predation by cursorial predators (principally lion, leopard and hyaena) is a serious problem for baboons [3,22,23], especially at night when these predators are most active and primates are at their most vulnerable because of poor night vision. Indeed, very small social groups are rare among baboons (groups < 15 comprise just 11% of our sample), and when they occur will often fuse with neighbours in order to be above the minimum size for local predation risk [24].

The processes that underpin this pattern (predation risk and infertility driven by group-living) are factors that all mammals have to contend with. As a negative relationship between fertility and group size seems to be widespread among mammals ([14]; see the electronic supplementary material), it seems likely that the present results will extrapolate to most large cursorial mammals. A fertility constraint might well explain why the most common social formations are either small harem-like groupings (where the number of breeding females is limited) or large herds (where fission–fusion allows fertility costs to be defused). Whereas the latter represents a casual solution (animals can join and leave groups individually), we can expect the former (which typically comprise bonded groups [15]) to exhibit coupled oscillators similar to those described here.

## Supplementary Material

Dunbar etal Baboon group size ESM_BiolLetts

## Supplementary Material

Supplementary Datafile1

## Supplementary Material

Supplementary Datafile2

## Data accessibility

The data are provided in the electronic supplementary material.
