# Supplementary material for: Trade-off between fertility and predation risk drives a geometric sequence in the pattern of group sizes in baboons
Source: Biol Lett. 2018 Mar 7;14(3):20170700. doi: 10.1098/rsbl.2017.0700 (PMC5897608; doi:10.1098/rsbl.2017.0700)
Supplement: Dunbar etal Baboon group size ESM_BiolLetts [file rsbl20170700supp1.docx]

**The Tradeoff Between Fertility and Predation Risk Drives a Geometric Sequence in the Pattern of Group Sizes in Baboons**

**R.I.M. Dunbar, Padraig Mac Carron, Cole Robertson**

**Supplementary Information**

***Supplementary Methods***

We take group (or troop) size to be that defined by the field worker: in these species, this grouping is fairly obvious, since the baboons forage and sleep together in stable social groups that maintain a degree of demographic coherence and stability over time as well as spatial separation from neighbouring groups.

All group sizes are either based on censuses on a particular date or an average across a study period, as specified in the original study. In populations that were subject to long term study (more than a single year) with repeated censuses of the same social groups, we counted a census as being new only providing it had been carried out at least five years after the previous census of that group. In either case, we always used the original (i.e. earliest) census and at most one later census. The data are provided in *Supplementary Dataset1.*

We used maximum-likelihood methods (Clauset et al. 2009) to fit a set of common distributions (power law, truncated power law, geometric, negative binomial, exponential, stretched exponential, normal, lognormal, and compound Poisson distributions of different magnitudes), using the discrete approach as described by Clauset et al. (2009). We numerically maximised the log-likelihood of each candidate distribution to obtain its parameter estimates, using the *optimize* module of Python's *scipy* (v0.17.1) library. We identified the most likely model using AIC. In order to choose the optimal number of clusters, we calculated a goodness of fit for each number of clusters and take a value of 0.85 as the threshold (following Coulson 1987). Because models with more parameters are always more likely to fit the data, we applied the Jenks natural breaks clustering algorithm (Jenks 1967) to see if a different approach gives the same result.

In most cases, fertility rates are based on observed mean birth rate or mean interbirth interval for a single social group, as specified in the original publications. For the two Guinea baboon (*Papio papio*) samples, birth rates were estimated from the number of immatures per female, averaged across several groups, for each of the two study sites (Boese 1975, pers. comm.; Sharman 1981). In each case, a group was censussed just once, with the sample based on a large number of troops (13 and 17). Immatures are defined as animals that are pre-puberty, with puberty occurring at around 4 years of age (Altmann et al. 1977). Juvenile mortality will inevitably lead to some underestimate of actual birth rates in this case; however, since both newborns and older juveniles are included, the degree of underestimate will be lessened since only the latter can have experienced significant mortality. Moreover, other studies indicate a good correlation between fertility measured in this way and actual birth rates (Dunbar 2017). It should be noted, however, that we do not rely on these particular datapoints to determine the pattern observed in baboon fertility, but rather include them for completeness so as to be able to place *P. papio* into the comparative context. The pattern remains the same whether or not we include the *P. papio* data. The data are given in *Supplementary Dataset2*.

***Supplementary Results***

*Group size*

Table S1 gives the cluster means (and the mean scaling ratio between successive clusters) determined by the two clustering methods. These are in close agreement. The MLE data are based on the four Poisson distributions identified as the best fit to the data (see Table 1). The Jenks algorithm also identified four clusters as optimal. In both cases, the scaling ratio approximates 2, indicating that it is likely to be underpinned by some kind of binary fission process.

*Table S1. Cluster means and mean scaling ratios between successive clusters generated by the two algorithms.*

Species Cluster means Mean

scaling ratio

MLE 18.2 38.8 75.4 168.2 2.10

Jenks 19.9 45.4 85.5 183.2 2.10

Mean 19.1 42.1 80.5 175.7 2.10

Number of groups* 202 132 61 14

* Number of groups assigned to the cluster by the algorithms.

Fig. S1 plots the distribution of the four theoretical clusters against the actual distribution of group sizes.


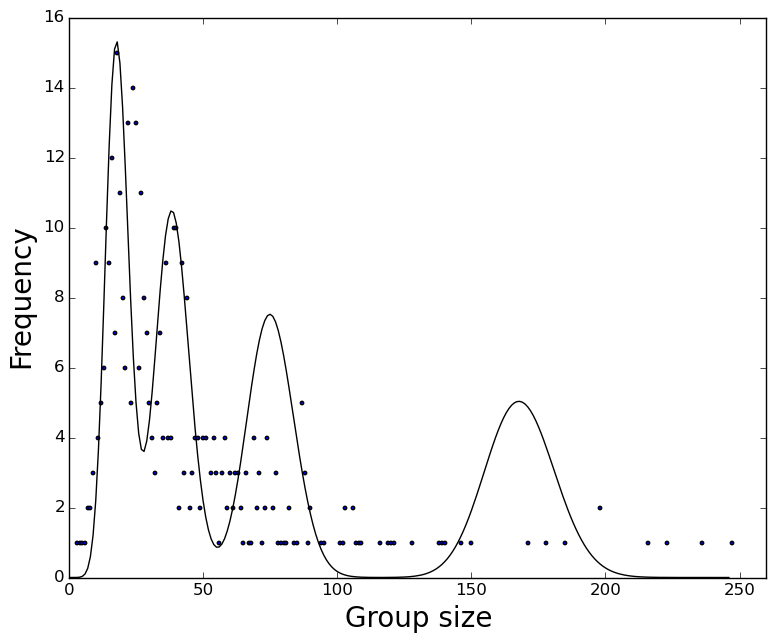


*Figure S1*

*Distribution of individual group sizes, overlain by the four theoretical cluster distributions generated by the four Poisson distributions produced by the MLE analysis.*

We checked the distribution of group sizes for each baboon species separately (Table S2). The Jenks algorithm identified either three or four clusters as optimal in each case, with cluster means that are very close to those observed in the sample as a whole

(Table S1). Note that, in the case of *P. ursinus*, the means for clusters 3 and 4 actually straddle the overall mean for cluster 3 for the sample as a whole, with a combined mean of 80.4 (N=18) and a mean scaling ratio of 2.13.

These data confirm that a pattern of four clusters is characteristic of all four species with broadly similar mean values (albeit with some species missing the smallest or largest cluster), and is not simply a difference between species. *Papio anubis* does not have any groups in the largest cluster (~160) and *Papio papio* lacks groups in the smallest cluster (~20). All have scaling ratios between successive cluster means of ~2.

*Table S2. Optimal number of clusters, and the resulting cluster means and mean scaling ratios between successive clusters, for each of the species separately, compared to that for the pooled sample, using the Jenks algorithm.*

Species Cluster means * Mean

scaling ratio

**All *Papio* 19.9 (202) 45.4 (132) 85.5 (61) 183.2 (14) 2.10**

*P. anubis* 22.2 (58) 49.3 (33) 94.1 (13) 2.06

*P. cynocephalus* 20.7 (32) 46.6 (41) 80.0 (28) 176.0 (4) 2.06

*P. ursinus* 17.8 (99) 37.1 (49) 64.9 (10) 99.8 (8) 1.79

*P. papio* 42.0 (19) 113.0 (9) 216.3 (6) 2.30

* Numbers in parentheses in the body of the table are the number of groups assigned to each cluster by the algorithm.

*Population-specific oscillator preferences*

To determine whether individual populations have groups in all oscillators or exhibit a preference for one oscillator over the others, we first determined whether the distribution of group sizes at each site was normally distributed or not. Of the 25 sites with a sample large enough to test, 20 were normally distributed (Kolmogorov-Smirnov one-sample tests against a normal distribution, p>0.05). In general, then, most sites have a characteristic group size with a single modal value. Of the 5 non-normal populations, two (Giant’s Castle and Nairobi Park) were bimodal within the same oscillator; the other three (Amboseli Park, Gilgil and Mt Assirik) were all essentially unimodal with a very small number of outliers with >100 animals. If groups >100 are excluded, these three are all normally distributed around a single modal value.

We then ran a *k-*means cluster analysis (with *k*=2) on each population for which at least 5 groups had been sampled, and determined the number of groups that fell into each cluster. In 13 cases, the analysis yielded a partition into 20/40 and 40/80, while in five cases the partition was 40/80 and 80/120, with three (Kimana, Mikumi and Okavango) having a 20/40 and 80/160 split. In an additional 7 cases, the analysis forced the distribution into two clusters *within* the same oscillator (always the 20-40 oscillator). Figure S2 plots the proportion of groups in the smaller (20-40) oscillator in each population. The data fall naturally into two clusters: those with most or all their groups in the 20/40 oscillator and those with most or all of their groups within the 40/80 or 80/120 oscillators. No populations exhibit a completely even split. Indeed, both subsets have the kind of bimodal distribution that would be expected from the stickiness of an imperfect statistical process: some groups from the 20/40 oscillator set take longer to fission than they ideally should, and some groups in the 40/80 oscillator partition unequally and sometimes produce daughter groups that are smaller than ideal.


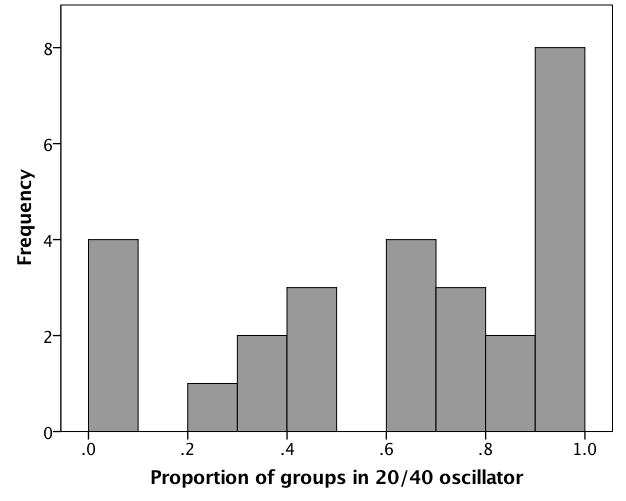


*Figure S2*

*Proportion of groups that fall into the lower (20-40) oscillator for 19 populations with N>4 groups sampled. Source: ESM Dataset S1*

*Identifying the phase transition*

In order to establish whether, and exactly where, there is a phase transition in the distribution of group sizes in Fig. 1(b), we calculated the goodness of fit (χ^2^) for the number of datapoints below and above a group size of 40 at different rainfall cutoff values. Fig. S3 plots the respective values of χ^2^. Only cutoffs between approximately 850-1090mm yield a significantly non-random distribution; that at 1000mm yields a clear maximum, suggesting that ~1000mm identifies a phase transition.


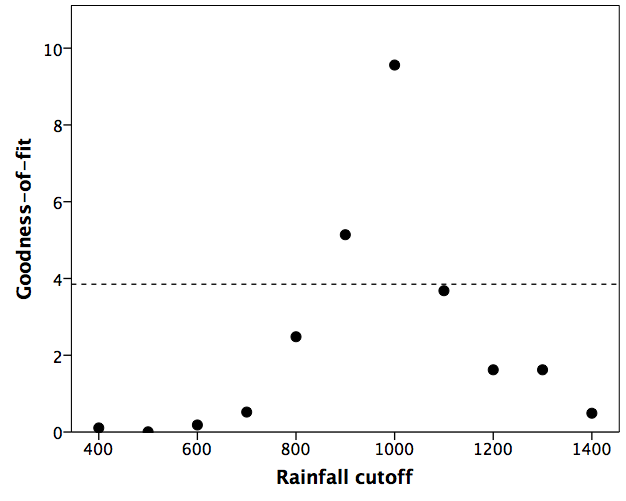


*Figure S3*

*Goodness-of-fit of the datapoints in Fig. 1(b) to a random distribution with the observed proportion below versus above a group size of 40 as a function of different rainfall cutoff values. The horizontal dashed line marks the minimum value of* χ^2^ for statistical significance (p=0.05 with df=1).

*Fertility*

The best-fit equation to the data in Fig. 2(a) is: b=0.097+0.0148N-0.000128N^2^ (where b = annual birth rate per female and N = group size) (F_2,13_=15.73, r^2^=0.708, p=0.0003; linear: F_1,14_=0.18, r^2^=0.012, p=0.682).

The quadratic relationship between birth rate and group size in Fig. 2(a) holds individually across the three species for which there are sufficient datapoints to run a regression. Although the regressions are not individually significant, all are quadratic in the same direction (*P. anubis*: F_1,2_=0.02, p=0.979; *P. cynocephalus*: F_2,2_=12.18, p=0.076; *P. ursinus*: F_2,3_=7.08, p=0.073; all linear regressions, 0.875≥p≥0.317). Taken together, the set of equations is significantly more positively quadratic than would be expected if there were no underlying trend (Fisher’s meta-analysis for a directional hypothesis: χ^2^=14.59, df=2x3=6, p=0.024).

Hill et al. (2000) reported that baboon fertility is independently predicted by both the number of adult females in the group and the mean ambient temperature of the habitat (an index of habitat quality). Mean annual temperature and group size are not correlated in the fertility sample (r_s_=-0.10, N=15, p=0.723). Nonetheless, to check whether environmental conditions might be a confound in our results, we regressed birth rate on mean habitat ambient temperature as a quadratic relationship (b = -0.672 + 0.104Temp - 0.00231Temp^2^: F_2,18_=3.77, r^2^=0.296, p=0.043; linear: F_1,19_=0.01, r^2^=0.000, p=0.933), and calculated residual birth rates from this regression. The results (Fig. S4) are identical to those shown in Fig. 2(a).


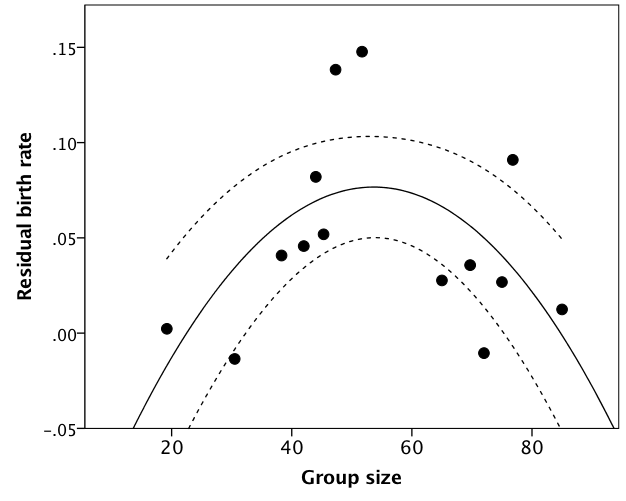


*Figure S4*

*Residual of mean birth rate regressed on mean local temperature plotted against group size for individual baboon groups.*

*Table S3. Backwards stepwise regression analysis of birth rate as a function of group size and temperature and their respective squared values.*

Model parameters r^2^ _adj_ F df p AICc

-----------------------------------------------------------------------------------------------------------

Group + Group^2^ 0.654 13.31 2,11 0.001 -132.07

Group + Group^2^ + Temp 0.621 8.10 3,10 0.005 -129.63

Group + Group^2^ + Temp + Temp^2^ 0.626 6.45 4,9 0.010 -127.03

-----------------------------------------------------------------------------------------------------------

It is worth noting that a backwards stepwise regression with both group size and temperature, and their squares, yields a best fit equation that has only group size and its square. The next best fit adds a linear effect of temperature, but the gain in fit is negligible (Table S3). More importantly, a model comparison procedure using AIC identifies a model with just group size and its square as a significantly (ΔAIC>2) better fit than either of the alternative models. In sum, group size seems to be a more important determinant of fertility than habitat quality.

*Fertility and group size in mammals*

There is evidence that fertility declines with social group size in a taxonomically very wide range of mammals, including zebra (Pluháček et al. 2006), most canids (Moehlmann & Hofer 1997), hyaena (Holecamp et al. 1996), badgers (Woodroffe & MacDonald 1995), rodents (Huck et al. 1988; Kinahan & Pillay 2008, Wey et al. 2013), marmots (Downhower & Armitage 1971), mongoose (Creel et al. 1992) and meerkats (Young et al. 2006), as well as domestic stock (McClure 1968). Experimental studies of domestic stock indicate that stocking rate (i.e. social group size) is mainly responsible for this, independently of any impact that nutrition might have (von Borell et al. 2007; Einarsson et al. 2008; Dobson et al. 2012; Clarke 2014). Since the crucial factor underpinning the oscillator mechanism seems to be widespread in mammals, it seems reasonable to expect that the same effects that we observe in baboons will be found in other mammalian taxa.

*Optimal transition point between oscillators*

We model the reproductive payoffs that accrue when switching oscillator at different group sizes across the range of group sizes of interest (20-80 individuals). To do this, we use an average reproductive lifespan (age at first birth to age at maternal death) of 13 years (based on Bronikowski et al. 2002; Altmann & Alberts 2003; Cheney et al. 2004). Alternative choices of length of reproductive lifespan do not change the payoff ratios. We considered different pairs of oscillators across the range of group sizes 20-80 individuals, using switch points between the two oscillators varying between 25-75 in steps of 5. This gives us successive pairs of oscillators: 20-25 vs 25-80, 20-30 vs 30-80, 20-35 vs 35-80, etc. For each pair of oscillators, the female starts in a group of the lower value (i.e. for the 20/25 vs 25/80 pair, the female starts in a group of either 20 or 25, while in the 20/30 vs 30/80 pair she starts in a group of 20 or 30). We summed the likelihood that a female would produce an infant (from Fig. 2a) in each successive year from the starting group size, allowing for year-by-year growth in group size as a result of births. For computational convenience, we assume that adult females account for 30% of total group size (a value that is typical of primates in general [Dunbar et al. 2018] and baboons in particular [Dunbar & MacCarron, in press]). For present purposes, we ignored mortality; since there is no evidence that this is size-dependent, its effect will be constant and, as with all good modelling, no advantage is gained by making the model unnecessarily complicated by including it. When group size hits the ceiling defined by a given oscillator, the group undergoes fission and the female’s group reverts to the oscillator baseline (unless the ceiling is below 20, in which case in resets to 50% of the size at fission) since this is the smallest daughter group into which a group can partition and thus represents the worst case scenario; this cycle was repeated until the female ‘died’. Our index is then the ratio of payoffs to a female under the two conditions (i.e. oscillators). To make the presentation of the results more intuitive, we always use the larger payoff (fitness) as the denominator.

***REFERENCES***

Altmann, J. & Alberts, S.C. (2003). Variability in reproductive success viewed from a life‐history perspective in baboons. *American Journal of Human Biology* 15: 401-409.

Altmann, J., Altmann, S.A., Hausfater, G. & McCuskey, S.A. (1977). Life history of yellow baboons: physical development, reproductive parameters, and infant mortality. *Primates* 18: 315-330.

Boese, G.K. (1975). Social behaviour and ecological considerations of West African baboons (*Papio papio*). In: R.H. Tuttle (ed.) *Socioecology and Psychology of Primates*, pp. 205-230. The Hague: Mouton.

von Borell, E., Dobson, H. & Prunier, A. (2007). Stress, behaviour and reproductive performance in female cattle and pigs. *Hormones and Behavior* 52: 130-138.

Bronikowski, A.M., Alberts, S.C., Altmann, J., Packer, C., Carey, K.D. & Tatar, M. (2002). The aging baboon: comparative demography in a non-human primate. *PNAS* 99: 9591–9595.

Cheney, D.L., Seyfarth, R.M., Fischer, J., Beehner, J., Bergman, T., Johnson, S.E., Kitchen, D.M., Palombit, R., Rendall, D. & Silk, J.B. (2004). Factors affecting reproduction and mortality among baboons in the Okavango Delta, Botswana. *International Journal of Primatology* 25: 401-428.

Clarke, I.J. (2014). Interface between metabolic balance and reproduction in ruminants: Focus on the hypothalamus and pituitary. *Hormones and Behavior* 66: 15-40.

Clauset, A., Shalizi, C.R., & Newman, M.E.J. (2009). Power-law distributions in empirical data. *SIAM Review* **51**, 661-703.

Clutton-Brock, T.H., Gaynor, D., McIlrath, G.M., Maccoll, A.D.C., Kansky, R., Chadwick, P., Manser, M., Skinner, J.D. & Brotherton, P.N.M. (1999), Predation, group size and mortality in a cooperative mongoose, *Suricata suricatta*. *Journal of Animal Ecology* 68: 672–683.

Coulson, M.R. (1987). In the matter of class intervals for choropleth maps: with particular reference to the work of George F. Jenks. *Cartographica* **24**, 16-39.

Creel, S., Creel, N., Wildt, D.E., & Monfort, S.L. (1992). Behavioural and endocrine mechanisms of reproductive suppression in Serengeti dwarf mongooses. *Animal Behaviour*, 43, 231-245.

Dobson, H., Fergani, C., Routly, J.E. & Smith, R.F. (2012). Effects of stress on reproduction in ewes. *Animal Reproduction Science* 130: 135-140.

Downhower, J.F., & Armitage, K.B. (1971). The yellow-bellied marmot and the evolution of polygyny. *American Naturalist*, 105, 355-370.

Dunbar, R.I.M. & Nathan, M. (1972). Social organisation of the Guinea baboon, *Papio papio*, in Senegal. *Folia Primatologica* 17: 321-334.

Dunbar, R.I.M., Korstjens, A.H. & Lehmann, J. (2009). Time as an ecological constraint. *Biological Reviews* 84: 413-429.

Dunbar, R.I.M., MacCarron, P. (in press). Group size and social structure in baboons reflects a trade off between fertility and predation risk. *Journal of Human Evolution*.

Dunbar, R.I.M., MacCarron, P. & Shultz, S. (2018) Primate social group sizes exhibit a regular scaling pattern with natural attractors. *Biology Letters* (in press)*.*

Einarsson, S., Brandt, Y., Lundeheim, N. & Madej, A. (2008). Stress and its influence on reproduction in pigs: a review. *Acta Veterinaria Scandinavica* 50: 1-48.

Hill, R.A., Lycett, J. & Dunbar, R.I.M. (2000). Ecological determinants of birth intervals in baboons. *Behavioural Ecology* 11: 560-564.

Holekamp, K.E., Smale, L., & Szykman, M. (1996). Rank and reproduction in the female spotted hyaena. *Journal of Reproduction and Fertility*, 108, 229-237.

Huck, U.W., Lisk, R.D., & McKay, M.V. (1988). Social dominance and reproductive success in pregnant and lactating golden hamsters (*Mesocricetus auratus*) under seminatural conditions. *Physiology and Behavior*, 44, 313-319.

Kinahan, A.A., & Pillay, N. (2008). Dominance status influences female reproductive strategy in a territorial African rodent *Rhabdomys pumilio*. *Behavioral Ecology and Sociobiology*, 62, 579.

McClure, T.J. (1968). Malnutrition and infertility of cattle in Australia and New Zealand. *Australian Veterinary Journal* 44: 134-138.

Moehlman, P.D., & Hofer, H. (1997). Cooperative breeding, reproductive suppression, and body mass in canids. In N.G. Solomon & J.A. French (eds.) *Cooperative breeding in mammals* (pp. 76-128). Cambridge, UK: Cambridge University Press.

Patzelt, A., Zinner, D., Fickenscher, g., Dieghou, S., Camara, B., Stahl, D. & Fischer, J. (2011). Group composition of Guinea baboons (*Papio papio*) at a water place suggests a fluid social organization. *International Journal of Primatology* 32: 652-668.

Pluháček, J., Bartoš, L., & Čulík, L. (2006). High-ranking mares of captive plains zebra Equus burchelli have greater reproductive success than low-ranking mares. *Applied Animal Behaviour Science*, 99, 315-329.

Sharman, M. (1981). *Feeding, Ranging and Social Organization of the Guinea Baboon*. PhD thesis, University of St. Andrews.

Wey, T.W., Burger, J.R., Ebensperger, L.A., & Heyes, L.D. (2013). Reproductive correlates of social network variation in plurally breeding degus (*Octodon degus*). *Animal Behaviour*, 85, 1407-1414.

Woodroffe, R., & MacDonald, D.W. (1995). Female/female competition in European badgers Meles meles: effects on breeding success. *Journal of Animal Ecology*, 64, 12-20.

Young, A.J., Carlson, A.A., Monfort, S.L., Russell, A.F., Bennett, N.C. & Clutton-Brock, T. (2006). Stress and the suppression of subordinate reproduction in cooperatively breeding meerkats. *Proceedings of the National Academy of Sciences*, *USA*, 103: 12005-12010.
